# What we thought we knew about TNF—And what we now must re-learn

**DOI:** 10.1016/j.stemcr.2026.102827

**Published:** 2026-02-26

**Authors:** Alexandra Rundberg Nilsson

**Affiliations:** 1 Lund Stem Cell Center, Lund University, Lund, Sweden; 2 Division of Molecular Medicine and Gene Therapy, Institution for Laboratory Medicine, Lund University, Lund, Sweden

## Abstract

Tumor necrosis factor (TNF) was long cast as a hematopoiesis villain, driving bone marrow and hematopoietic stem cell (HSC) suppression. We now see that TNF’s effects are cell type-, context-, and time-dependent. Rather than being simply “bad,” TNF can prune progenitors while transiently reprogramming HSCs without sacrificing long-term regenerative capacity.

## The early view—“TNF is bad for HSCs”

The view that TNF is bad for hematopoietic stem cells (HSCs) is rooted in early *in vivo* and *in vitro* studies showing that TNF suppresses colony formation and regenerative capacity in human and murine hematopoietic stem and progenitor cells (HSPCs) (Schuettpelz and Link, 2013). However, some reports also noted that the inhibitory effects disappeared when TNF was washed out before culturing, suggesting that its impact could be reversed. Moreover, conflicting findings emerged, describing proliferative, survival-promoting, or even increased regenerative effects on primitive cells under certain cytokine conditions or at baseline (Abboud et al., 1987; Rezzoug et al., 2008; Rusten et al., 1994). Still, the prevailing conclusion for decades was simple: TNF is harmful for primitive hematopoiesis, and its elevation inherently damaging.

## Cracks in the dogma—Context and cell-type matter

While early work emphasized TNF’s suppressive effects, later studies have revealed that its impact depends heavily on both cell type and context. TNF is capable of triggering distinct intracellular programs: apoptosis or necrosis via TRADD/FADD/RIPK1/3, or survival and proliferation through NF-κB signaling (Brenner et al., 2015). This dual potential, well established in other cell types, became highly relevant for HSC biology when Yamashita and colleagues systematically dissected TNF effects across hematopoietic compartments *in vitro*. Their study marked a turning point: TNF induces apoptosis in myeloid progenitors, yet protects HSCs by inhibiting necroptosis and promoting myeloid regeneration through NF-κB-dependent programs (Yamashita and Passegue, 2019).

Our own *in vivo* findings supported this compartment-specific model. Following acute TNF exposure, HSC numbers in the bone marrow increase, while committed progenitors from all major lineages (lymphoid, myeloid, megakaryocytic, and erythroid) decline (Rundberg Nilsson et al., 2023). Yamashita et al. further demonstrated that the outcome of TNF stimulation changes depending on the cytokine composition in culture (Yamashita and Passegue, 2019), reinforcing earlier work showing that extracellular, in addition to intracellular, context shapes TNF’s net effect. Together, these findings show that TNF is not universally cytotoxic for all primitive hematopoietic cells, but acts in a cell-type- and context-dependent manner.

This compartment-specific sensitivity helps reconcile previous conflicting results. When readouts are dominated by progenitors, TNF appears suppressive; when enriched for HSCs, protective effects may emerge. Transplantation assays using unpurified populations, with progenitor loss but HSC preservation increases relative HSC frequencies, further confounding interpretation. Interpretations may also be misleading with respect to peripheral output potential when cell populations are quantified solely by relative frequencies rather than absolute numbers or concentrations. Moreover, in differentiation cultures, continuous TNF exposure reduces total yields because progenitors undergo apoptosis as HSCs differentiate into them, giving the false impression that HSCs themselves are lost.

## Adding the time dimension—TNF effects can be reversible

**Figure 1:**
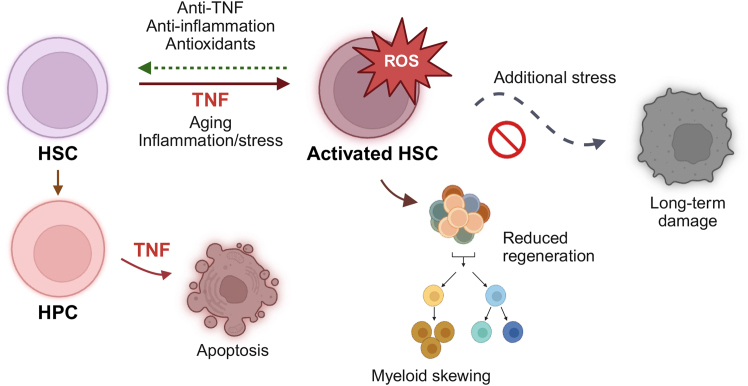
TNF effects on hematopoiesis are cell-, context-, and time dependent TNF selectively induces apoptosis in hematopoietic progenitors, while HSCs are protected and transiently activated. During this activated state, HSCs display reduced regenerative capacity, myeloid-biased output, and elevated ROS levels—features also observed during aging and in response to other inflammatory and cellular stressors. Additional stress during this state can cause permanent damage, however; the state may also be reversed upon withdrawal of TNF or through anti-inflammatory and antioxidant interventions.

TNF effects on HSCs are also highly time-dependent. Acute TNF exposure is followed by a transient loss of HSC quiescence, reduced reconstitution capacity, and myeloid-skewed output (Figure 1). However, when a recovery period is introduced before functional assessment, these functional deficits are reversed, both after a single dose of TNF and more prolonged exposure (Rundberg Nilsson et al., 2023). Similar reversibility has been documented with other cytokines like interleukin-1 (IL-1) (Pietras et al., 2016), indicating that inflammatory stress can impair HSC function acutely but does not necessarily cause permanent damage if the stimulus is removed. These works emphasize that a snapshot measurement taken during or immediately after inflammatory insult can substantially underestimate long-term HSC resilience. Nevertheless, additional stress applied before the HSCs have recovered may accumulate damage, metabolic strain, or selection pressures that lead to long-term dysfunction or clonal shifts (Caiado et al., 2021; Pietras et al., 2016).

## Targeting inflammation to preserve hematopoietic function

Our work further demonstrated that transient TNF blockade in transplant recipients improves donor HSC engraftment (Rundberg Nilsson et al., 2023), potentially by counteracting TNF surges during conditioning. Consistent with this, inflammatory donor profiles, including elevations in TNF, associate with impaired engraftment (Sobrino et al., 2025). Donor antioxidant treatment can mitigate TNF-induced reactive oxygen species (ROS) production, preserving HSC reconstitution potential under inflammatory stress (Ishida et al., 2017), and accelerating hematopoietic recovery and early engraftment (Wang et al., 2017). Moreover, inhibition of TNF-driven ERK/ETS1/IL27Ra signaling can rejuvenate hematopoietic aging (He et al., 2020), which is also observed following anti-IL-1 and antioxidant treatments (Capitano et al., 2021; Kovtonyuk et al., 2022).

Hematopoietic phenotypes seen in inflammation, stress exposure, and aging are mechanistically linked: proinflammatory cytokines such as TNF and IL-1 activate stress-response pathways that elevate ROS production, skew lineage output toward increased myeloid cells, and reduce regenerative capacity. However, sustained exposure to TNF and other inflammatory stimuli, as occurs during aging or chronic infection and disease, may drive clonal remodeling that contributes to reduced regeneration and myeloid-biased output through mechanisms that are distinct from those engaged by acute exposure. It is plausible that both the net hematopoietic effect and the capacity to recover are determined not only by the type of inflammatory or infectious stimulus, but also its duration and dose. Nevertheless, interventions targeting this inflammatory axis—whether through anti-TNF, anti-IL-1, or antioxidant strategies—offer means to protect HSC function, enhance transplantation outcomes, and limit or even reverse cumulative damage over a lifetime.

## Implications for HSC research

The old story was simple: TNF is bad for hematopoiesis. The new story is more sophisticated: TNF’s effects are cell type-specific, shaped by intracellular and extracellular cues, and temporally reversible. This shift highlights a broader lesson for inflammation and stress biology: the importance of time and context in interpreting insult effects. Assessing HSC function too soon after inflammatory/stress exposure risks underestimating their long-term regenerative capacity, while lumping progenitors and stem cells together can obscure cell-specific effects. In addition, inflammatory mediators engage in multiple downstream pathways that are impacted by internal and external factors, and shift with cell state, age, and disease. The TNF story ultimately serves as a reminder that even long-standing dogmas must be revisited as new tools, higher-resolution analyses, and longitudinal approaches reveal a more complex truth.

## Acknowledgments

A.R.N. is supported by grants from The Swedish Research Council, Gunnar Nilsson’s Cancer Foundation, Åke Wiberg's Foundation, and Magnus Bergvall's Foundation. The figure was created in BioRender. Rundberg Nilsson, A. (2026) https://BioRender.com/jfep1xu.

## Declaration of interests

The authors declare no competing interests.
